# Tip-Enhanced Raman Imaging and Nano Spectroscopy of Etched Silicon Nanowires

**DOI:** 10.3390/s131012744

**Published:** 2013-09-25

**Authors:** Nastaran Kazemi-Zanjani, Erwan Kergrene, Lijia Liu, Tsun-Kong Sham, François Lagugné-Labarthet

**Affiliations:** Department of Chemistry and Centre for Advanced Materials and Biomaterials, University of Western Ontario, 1151 Richmond Street, London, N6A 5B7, Canada; E-Mails: nkazemiz@uwo.ca (N.K.-Z.); e.kergrene@gmail.com (E.K.); ljliu@suda.edu.cn (L.L.); tsham@uwo.ca (T.-K.S.)

**Keywords:** tip-enhanced Raman spectroscopy, atomic force microscopy, silicon nanowires, strain/stress-induced broadening

## Abstract

Tip-enhanced Raman spectroscopy (TERS) is used to investigate the influence of strains in isolated and overlapping silicon nanowires prepared by chemical etching of a (100) silicon wafer. An atomic force microscopy tip made of nanocrystalline diamond coated with a thin layer of silver is used in conjunction with an excitation wavelength of 532 nm in order to probe the first order optical phonon mode of the [100] silicon nanowires. The frequency shift and the broadening of the silicon first order phonon are analyzed and compared to the topographical measurements for distinct configuration of nanowires that are disposed in straight, bent or overlapping configuration over a microscope coverslip. The TERS spatial resolution is close to the topography provided by the nanocrystalline diamond tip and subtle spectral changes are observed for different nanowire configurations.

## 1. Introduction

Owing to their superior electronic, optical, mechanical, thermal and chemical properties, silicon nanowires (SiNWs) have been investigated for a wide range of potential applications. Silicon nanowires exhibit excellent photo catalytic activity [1], and due to their direct path of charge transfer [2], they can be used as anode materials and improve the storage capacity of lithium ion batteries [3]. In the field of photovoltaics, SiNW-based solar cells can achieve efficient absorption of sunlight by using only 1% of the active material required in conventional solar cells [4]. Because of their poor thermal conductance, silicon nanowires can also act as efficient thermoelectric materials [5]. However, these physical properties are sensitive to the nanoscale variations in the structure of the nanowires. For example, it has been reported that surface roughness influences the thermoelectric performance of silicon nanowires [6]. Therefore it is essential to develop characterization techniques that can provide insight into the structure of the nanowires on a nanometer scale. Raman spectroscopy can serve as a powerful and sensitive characterization technique since it is sensitive to the crystal lattice structure. The shape and frequency shift of the Raman peaks may reveal useful information about the crystallinity, the amorphicity, the induced mechanical strain and even about the diameter of nanoscale silicon nanowires [7,8] as well as other semiconductor nanoscale objects [9]. Therefore, Raman spectra of silicon nanowires are of great value for understanding the properties of isolated nanowires [10].

Despite the fact that these studies provide very useful information about the silicon nanowires, they all lack specificity due to their limited spatial resolution. Measurements acquired from far-field conventional measurements are an average of the bulk properties that results in the loss of the spatial information and the knowledge of the distribution of defects [11]. In an attempt to surpass this problem, tip-enhanced Raman spectroscopy (TERS) opens up many new possibilities, including better spatial resolution and better surface sensitivity [12].

TERS is a promising approach to obtain chemical information with nanometer scale resolution beyond the diffraction limit of light and is promising for the study of materials and biomaterials [13–17]. Noticeably, TERS has been used successfully as a nanocrystallography technique for the study of a variety of crystalline nanomaterials including GaAs and BatiO_3_ single crystals [18,19], GaN and Ge single nanowires [20–22], or silicon/silicon oxyde structures [23]. Nevertheless, due to the enhancement of the longitudinal field by the metal tip [24] and the possible depolarization effects from the TERS probe, polarized TERS measurements are limited, unlike micro-Raman experiments where full polarization measurements are accessible for both excitation and scattered signals [11,25,26]. The diffraction limit of light implies that the accessible spatial resolution in an conventional optical measurement is limited to ∼λ/2, with λ being the wavelength of the probing radiation [27]. To date, TERS has reached a lateral spatial resolution of 10–15 nm, which provides significantly sharpened optical details of single carbon nanotubes deposited onto a surface [28]. In addition, due to a localized surface plasmon resonance (LSPR) which is confined at the apex of the metallic tip, TERS can provide detailed spectroscopic information from a functionalized surface or an interface with a better specificity. In TERS measurements, a tip made of metal (Au, Ag) or a commercial atomic force microscope tip made of Si, SiO_2_ or Si_3_N_4_ coated with a thin metal layer is brought into close proximity of a few nanometers from the sample. This tip, that acts as a nanoantenna [29], performs a point-by-point scan of the surface while its distance with the sample surface remains unchanged. A tightly focused laser beam with the proper polarization induces an excitation of the tip LSPR which in turn acts as a confined source of light. This source locally enhances the Raman scattering for each of the scanned points thus improves the spatial resolution and the surface specificity [30].

To our knowledge no previous TERS studies have been conducted on single silicon nanowires so far. In this work, isolated [100] nanowires have been dispersed onto a glass coverslip and studied under the TERS setup using a silver coated nanocrystalline diamond AFM tip. The spatial and spectral resolution accessible in the TERS setup allows for the precise assignment of the vibrational frequency shifts and the change of FWHM in silicon Raman signal to the strain induced in silicon nanowires due to the structural variations. It this study we have made use of nanocrystalline diamond tips that were coated with a silver layer to prevents the interference between the silicon signal of nanowires and the silicon signal from the most common silicon based AFM tips. Alternatively, other tips made of Si_3_N_4_, oxidized silicon or modified glass tips could potentially be used for similar studies [23].

## 2. Experimental Section

### 2.1. Sample Preparation

Silicon nanowires were synthesized following the method proposed by Zhang *et al.* [31]. N-type (phosphorous doped) silicon wafers with a thickness of 525 μm, a resistivity of 1–5 Ohm·cm and a (100) orientation were cut into smaller pieces and then cleaned with acetone, ethanol and water in a subsonic device. Briefly, the silicon pieces were first cleaned by immersing them for 10 min in a 3:1 (*v*/*v*) mixture of H_2_SO_4_ (98%) and H_2_O_2_ (35%) followed by immersion in a 5% HF solution for about 3 min. Silver nanoparticles (AgNPs) were then deposited on the wafer pieces by dipping the silicon wafers into a solution of 4.8 M HF and 0.005 M of AgNO_3_ for one minute and further washed with water to remove extra Ag^+^ ions. The silicon pieces with the uniform layer of nanoparticles were then etched in an etching solution composed of 4.8 M HF and 0.4 M H_2_O_2_ for 40 min. After the etching step, the samples were washed with water and the Ag catalyst particles were removed by immersion in a 1:1 (*v*/*v*) solution of HNO_3_ and H_2_O followed by an immersion in 5% HF solution. Finally the wafers were washed with water to remove the rest of corrosive HF from the wires. This results in single-crystal nanowires that are oriented normal to the surface of the (100) wafer, with [100] direction, with nominal diameter ranging from 40 to 200 nm and with lengths varying from 10 to 50 μm. To isolate and disperse the as-synthesized wires, the surface of the silicon wafer pieces was gently scratched by a razor blade and the scratched powder was transferred into ethanol. The resulting solution got homogenized ultrasonically for about 10 min and then a drop of this solution was transferred onto a clean glass microscope cover slide.

### 2.2. TERS Setup

**Figure 1.**
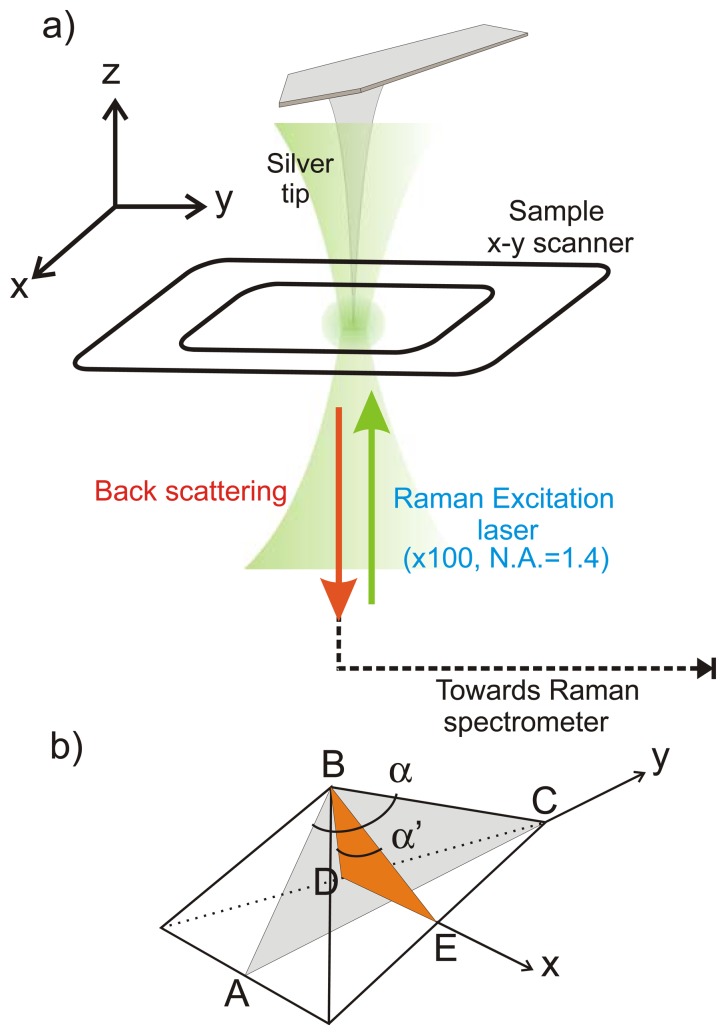
(**a**) Scheme of the TERS setup used for this study. Silicon nanowires are dispersed onto a glass coverslip and excitation light is linearly polarized along the x direction; (**b**) Geometry of the nanocrystalline tetrahedral diamond tip used for the TERS experiments. α = 125.24° and α' = 79.34°.

A Raman spectrometer (600 gr/mm grating, HR LabRam, Horiba-JobiYvon, Kyoto, Japan) connected to an inverted optical microscope (IX71, Olympus, Tokyo, Japan) was interfaced with an atomic force microscope (AFM, NanoWizard II Bioscience, JPK Instruments Inc., Berlin, Germany) to perform measurements in the back-scattering geometry as shown in Figure 1a. The AFM was equipped with a high resolution piezoelectric xy sample stage (TAO stage) as well as a xyz piezoelectric actuator to control independently the tip position. The linearly polarized incident laser beam (λ = 532 nm, Compass 315 M laser, Coherent, Santa Clara, CA, USA) was focused on the sample with a high numerical aperture oil immersion microscope objective (PlanAPO Olympus, N.A. = 1.4 × 100) which collects the backscattered light as well.

The power of the laser at the focal spot was set to about 300 μW. In this range of excitation intensity, no heating effect was observed. For this study, preliminary measurements of the Stokes and anti-Stokes Raman contributions were performed to rule out any possible thermal effects. For such low laser intensity, the Stokes/anti-Stokes intensity ratio was constant over the whole surface of the nanowire. Temperature effects were observed on silicon nanowires for power exceeding 500 μW. In such case the silicon signal was red shifted and the temperature increase was homogeneous over the whole nanowire. The TERS probe was attached to its own piezoelectric stage and was kept a few nanometers above the sample surface by means of a conventional AFM feedback mechanism. AFM topography were acquired in non-contact mode using either nanocrystalline diamond tips or silicon tips (f = 170 kHz, k = 48 N/m, NCL Nanoworld, Neuchatel, Switzerland). For TERS experiments nanocrystalline diamond AFM tips were exclusively used (ND-DTIRL-4 All-Diamond tip, typical oscillating frequency of f = 240 kHz and a force constant of k = 30 N/m, Advanced Diamond Technologies, Inc., Romeoville, IL, USA) and were coated with 5 nm of titanium followed by 20 nm of silver by means of electron-beam induced thermal evaporation of silver. The tetrahedral tip shows wide angles along the x and y direction, as depicted in Figure 1b. The coated tips were used within a day after coating before oxidation degrades the thin silver layer.

The input beam was polarized along the x direction as shown in Figure 1. When tightly focused with a high N.A. objective, the total electric field at the focal point is Gaussian in the transverse plane and has an elongated waist in the longitudinal direction. As previously reported by several groups [24,32,33], the z-component of the tightly focused input field shows two lobes oriented along the z axis. A critical point in TERS using a linearly polarized excitation source consists in placing the metalized tip in one of the two lobes so that the z polarized input light can excite the plasmon frequency of the metallic tip along the long axis of the tip oriented along z. The tip acts as nano-antennae for both reception of the EM input field as well for emission of the scattered field [29,30].

## 3. Results and Discussion

**Figure 2.**
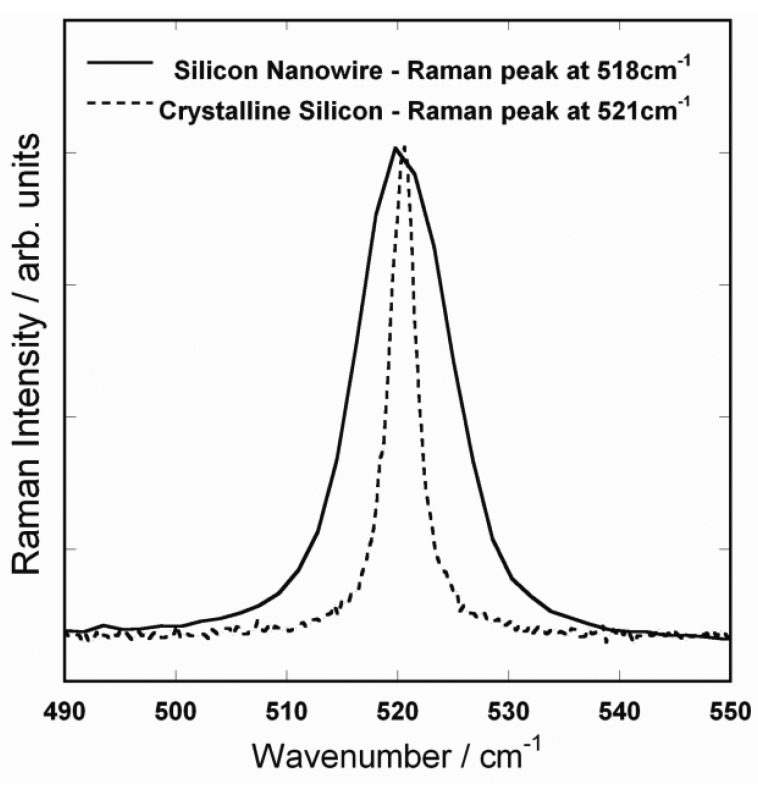
First order optical phonon Raman peak of silicon nanowire and (100) silicon wafer.

First, the Raman spectra of a single silicon nanowire and a bulk (100) silicon wafer were acquired (Figure 2). In the absence of internal or external stress, in back scattering geometry, the triply degenerate first order optical phonon modes of silicon (1 LO, 2TO) result in a single Raman peak at 521 cm^−1^ for a silicon wafer while it is shifted to 518 cm^−1^ for a 60 nm diameter silicon nanowire. Furthermore, the Raman peak shows a significant broadening with a full width at half maximum (FWHM) of 10 cm^−1^ as compared to 2.5 cm^−1^ for bulk silicon wafer. This spectral variations and a qualitative investigation of the phenomena was previously reported in literature [8]. In brief, when the crystalline size decreases, momentum conservation will be relaxed and Raman active modes will not be limited to be at the center of the Brillouin zone.

For silicon sample subject to stress, the characterization of the phonon modes (LO or TO) by Raman spectroscopy depends on the sample crystallinity, the orientation of the crystal with respect to the incident polarization but also the directivity of the stress (uniaxial or biaxial) and its sign (compressive or tensile). For negative uniaxial (compressive) stress along the [111] direction of a silicon wafer, the triply degenerate optical mode at q = 0 is split into two Raman peaks with wave vectors parallel (singlet) or perpendicular (doublet) to the strain direction [34,35]. In backscattering geometry, only the singlet can be observed and the optical-phonon frequency can be predicted with respect to the unstrained Si lattice [35].

**Figure 3.**
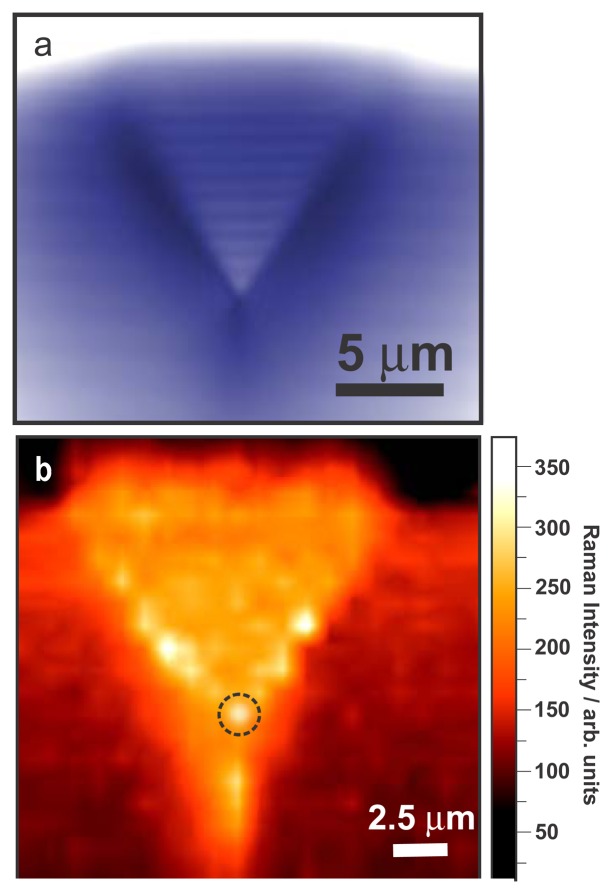

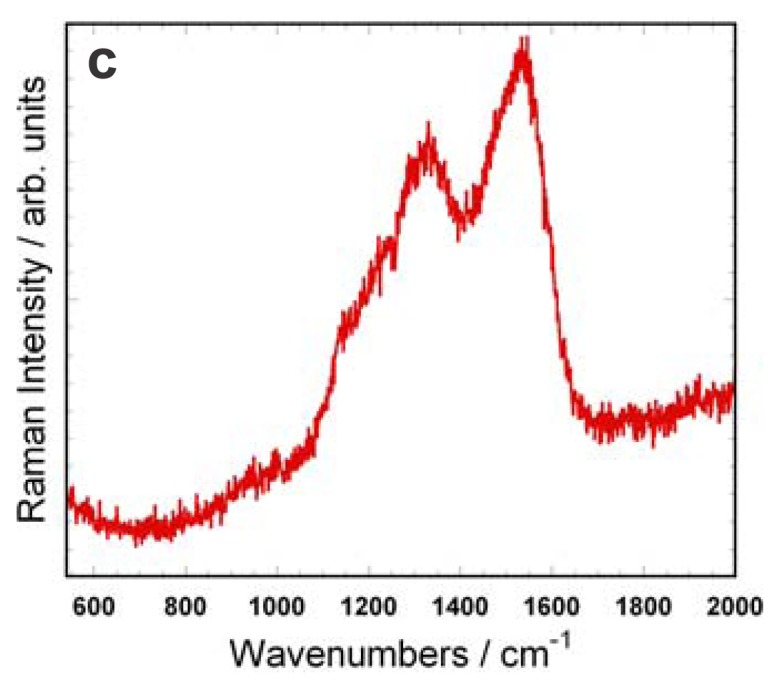
(**a**) Vertical deflection image of the nanocrystalline diamond tip and cantilever coated with a thin layer of titanium (5 nm) and silver (20 nm); (**b**) Raman intensity map of the same tip and cantilever. The three edges of the tetrahedral pyramid can clearly be distinguished; (**c**) Raman spectra of a nanocrystalline diamond tip coated with 20 nm of silver (adapted from [37]).

In TERS, the accessible spatial resolution depends mainly on the dimension of the tip apex which must be ideally positioned with respect to the irradiation source. Consequently, it is essential to determine the position of the tip apex in order to enable its alignment at the focal point of a tightly focused excitation laser. In this study, both vertical deflection images (Figure 3a) and Raman intensity maps of the silver-coated nanocrystalline diamond AFM cantilever (Figure 3b) have been utilized as a reliable method to find the position of the tip apex. The vertical deflection image (Figure 3a) and the Raman scattering of the tip (Figure 3b) both show clearly the tetrahedral structure of the tip. Scans of the tip over smaller area are then performed to optimize the signal and tune the position of the tip. Another approach using the Rayleigh scattering of tip can also be used to locate the tip rapidly and is complementary to vertical deflection and Raman imaging [36]. In the present study we could not record sharp Rayleigh scattering of the nanocrystalline diamond tip, presumably due to the wide angles formed by the tetrahedral tip of 125.24° and 79.34° along the Y and X axes (Figure 1b), respectively.

The Raman intensity map in Figure 3b is generated by integration of the Raman signal, using the 1,207–1,653 cm^−1^ spectral range scattered by the nanocrystalline diamond tip shown in Figure 3c. Prior to the TERS experiments, the tip cantilever was then immobilized at the defined x-y position in Figure 3b with the highest Raman intensity that corresponds to the tip position and laser focal point simultaneously. In Figure 3b one can observed some intense Raman spectra located along the sharp edges of the tetrahedral pyramid. More importantly, the shape of the Raman map together with the vertical deflection image also reveal clearly the tip position located at the crossing point of the three edges, allowing one to determine accurately the position of the tip. It should be noted here that the Raman signature of the nanocrystalline diamond would be different from a diamond material containing large diamond grains and rich in graphitic phase [38]. TEM observations on the nanocrystalline diamond have shown agglomeration of nanosize diamond grains with steep grain boundaries and no amorphous phase between them.

**Figure 4.**
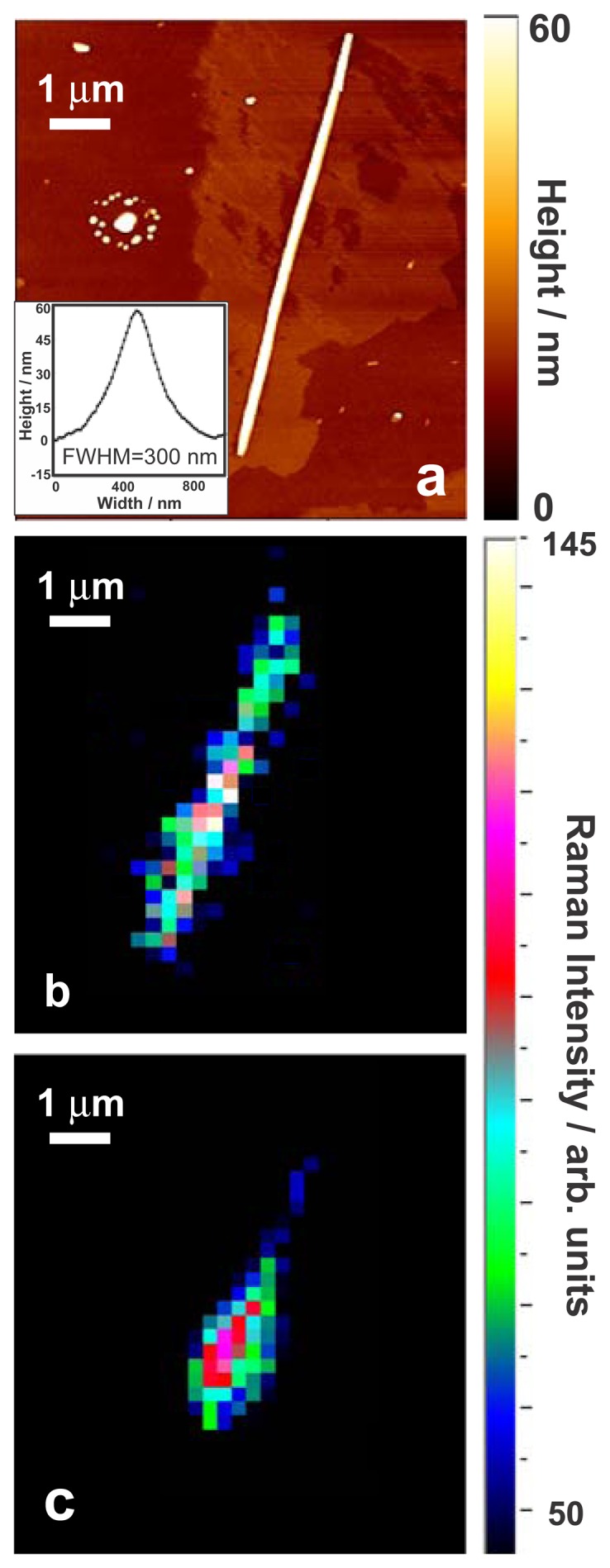
(**a**) AFM image and height cross section. The AFM image was done with a non-coated tip after the TERS experiments. Note that the lateral resolution of the AFM cross section is poor compared to the height resolution presumably due to the angles of the tip apex; (**b**) Total Raman intensity map (near + far field with tip in) showing the variation of average signal intensity of the silicon peak. The spectra were integrated over the 511–534 cm^−1^ range; (**c**) far-field (tip out) Raman average intensity map of a single silicon nanowire (the tip is withdrawn by 3 microns above the nanowire). For (**a**) and (**b**) the spectra were integrated over the 511–534 cm^−1^ range and an acquisition time of 1 s/spectrum was used.

After immobilization of the tip, preliminary AFM images of the nanowire were done in order to select points to be probed. Minimum interaction between the tip and the sample was used in order to avoid any wear of the metallic coating of the tip. This weak interaction may result in poorer spatial resolution of the TERS experiment. Sharper topographical images of the wire of interest were generally collected once the TERS measurements were finished using either the same tip or using a non-coated tip. TERS spectra of various silicon nanowires were collected in non-contact mode by moving the sample in the xy plane using a piezoelectric stage. AFM image along with the topographic cross section of a given silicon nanowire is presented in Figure 4a. This image was acquired after the TERS experiments were performed.

The height of the nanowire measured by AFM corresponds to the nominal diameter of the single nanowire (∼60 nm) while the lateral cross section of the wire shown as an inset of Figure 4a is about ∼300 nm due to the convolution with the geometry of the AFM tip. Raman spectra of the same nanowire were collected when the TERS tip was in proximity of the wire (tip in: far-field + near-field) and when the tip was withdrawn by 3 μm above the sample (tip out: far-field). The TERS signal is therefore the difference between the two collected spectra, *i.e.*, (tip in – tip out) to eliminate the far-field contribution. The Raman spectra were collected by step-scanning the isolated wire with a 250 nm step in the x and y direction. Smaller scan steps were first avoided to prevent the considerable increase of the map acquisition time. By integration of the first order optical phonon silicon Raman signal in the collected spectra, a Raman intensity map of the silicon nanowires were generated with 1- the tip close to the sample yielding the total signal that includes both near-field and far-field Raman contributions and with 2- the tip far from the sample yielding only to the far-field contribution. The maps are presented in Figure 4b,c, respectively. The acquisition time for collecting the individual Raman spectra was set to 1 s/spectrum. As it can be observed in the mapping shown in Figure 4b, a slightly better contrast is observed when the tip is closer to the sample even though pixel size was set to 250 nm and considering that this map still includes the far-field contribution. In Figure 4c the far-field mapping is less homogeneous implying that the near-field contribution is important to provide a good sensitivity to probe the full wire.

**Figure 5.**
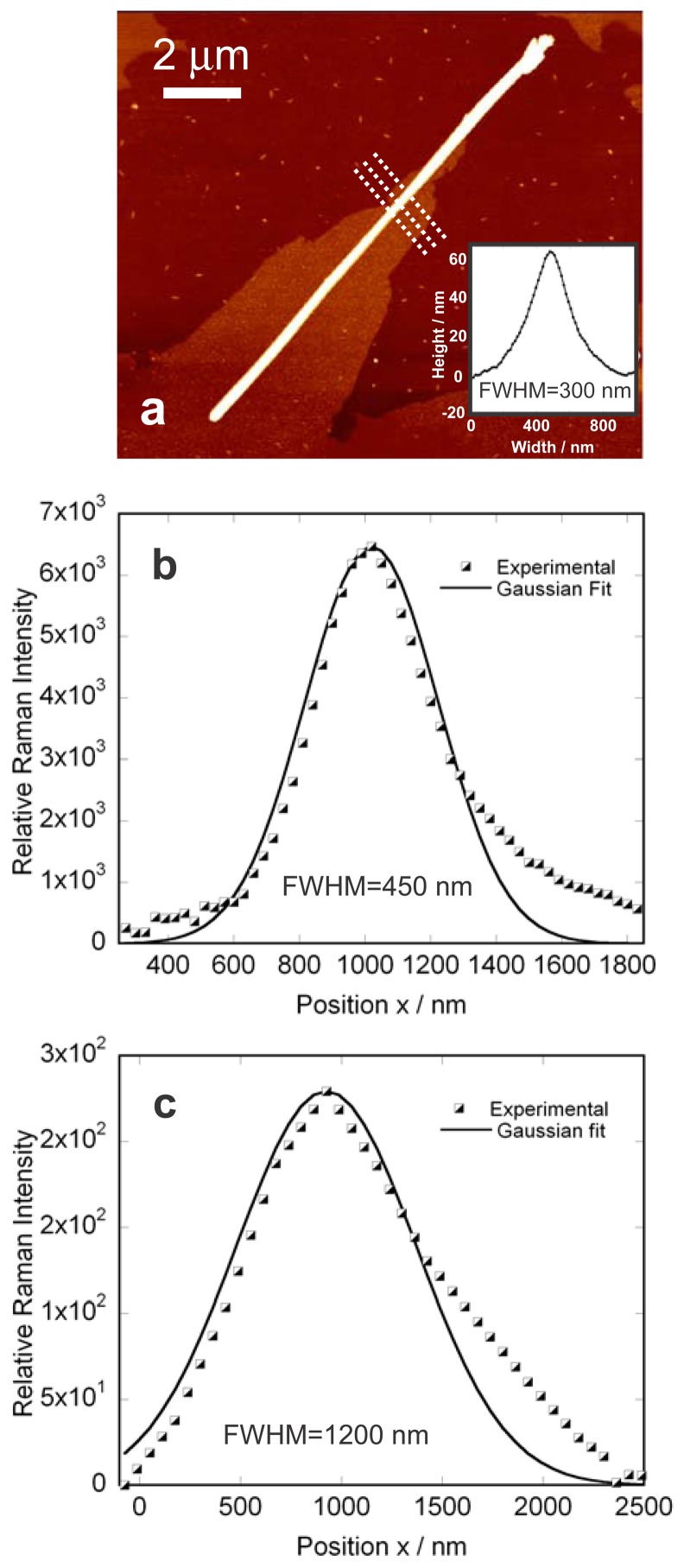
(**a**) AFM image of a single silicon nanowire and the topography cross section of the same nanowire. (Reproduced from [37]); (**b**) Total Raman signal when the metal tip is close to the sample. The full width at half maximum indicates a spatial resolution of ∼450 nm as compared to a topographical cross section of ∼300 nm as provided by the AFM. This signal includes the much weaker far-field (tip out) signal; (**c**) The optical cross section obtained in far-field measurements provides a full width at half maximum of 1,200 nm and a significantly weaker signal. For (**b**,**c**) an acquisition time of 15 s per spectrum was used.

As mentioned above, the relatively large size of the mapping area makes the collection process of the spectra lengthy if the separation between the individual points in the map decreases. On the other hand, thermal effects that might be induced during a long illumination process can reduce the reliability of the acquired results. Nevertheless, it is difficult to estimate the spatial resolution reached in the near-field measurements through analyzing the data acquired using 250 nm mapping steps along the nanowire due to the uncertainty caused due to the reflection of the laser from the edges of the wire. Furthermore the measurements acquired with the tip close to the sample also include the far-field contribution that significantly broadens the spatial resolution given the short acquisition time and low signal/noise ratio. For this reason, we have scanned three short lines on a random section of a nanowire with smaller step sizes and in the orthogonal direction with respect to the long axis of the wire. The acquisition time is now 15 s/spectrum. The lines are shown in Figure 5a. Each of the three lines were 3 μm long and the step between each individual spectra was set to 30 nm in both x and y direction along the lines. By integrating the silicon peak intensity for each pixel of the map and averaging the intensity values for corresponding pixels in these three lines, the spatial resolution limit of our experiment was estimated. Figure 5a shows the AFM image of the corresponding nanowire along with the topographic cross section curve. Variations in the intensity of the silicon Raman signal with tip in and tip out averaged along three lines are presented in Figure 5b,c. The two dashed curves in Figure 5b,c present the experimentally measured Raman intensities of the total field (near + far fields with tip in) and far-field (tip out) while the solid curves are the Gaussian model which is fitted on the experimental points. In this experiment, when the tip is close to the sample the signal is significantly higher (6,000 counts) compared when the tip is out (250 counts). The optical contrast defined by C = (S _tip in_−S _tip out_)/S_tip out_ would be in this case to ∼20. The determination of the local enhancement factor is more delicate since it implies a good knowledge of the volume of the tip that participates to the enhancement. A very broad range of values are generally reported in literature (enhancement factors varying from 10 to 10^4^) and the imprecision over the scattering focal volumes in the optical near- and far-fields is most probably the source of the reported broad values of enhancements [13].

The spatial resolution for this experiment was estimated by taking the average silicon peak intensity values in corresponding pixels of the three lines. As can be observed in Figure 5b, ∼450 nm resolution was obtained for near-field that matches the dimensions of the wire predicted by the topographic cross section curve (∼300 nm) more closely. Note that due to the difference of signal between the two experiments (tip in *versus* tip out), the removal of the far-field contribution from the experiment where the tip is closed to the sample does not improve the spatial resolution. We believe that any variation from the actual size of the wire is due to the geometry of the tip (wide angle) and possible the inhomogeneities of the coating. As shown in Figure 5c, the far-field map predicts a resolution of ∼1,200 nm which corresponds roughly to the dimension of the focal spot.

**Figure 6.**
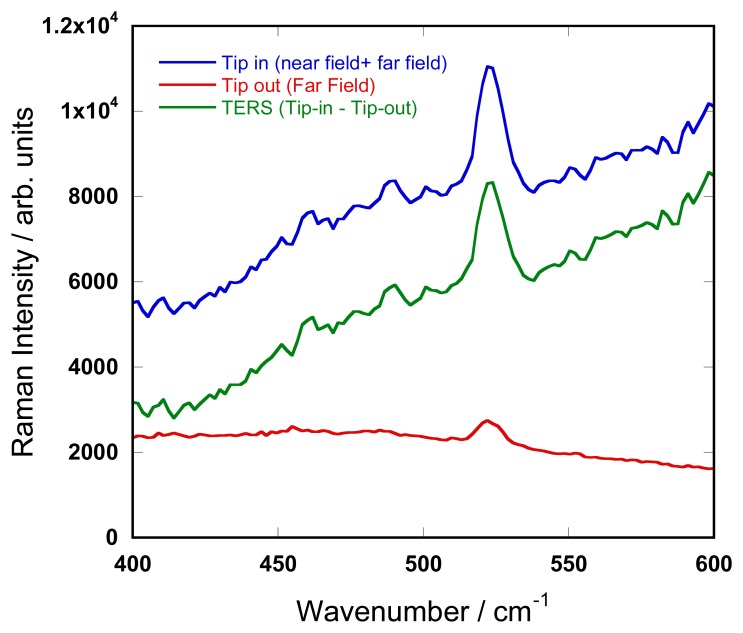
Raman spectra with metalized tip in positioned in the optical near-field of the sample (in feedback, tip in) and in the optical far-field of the sample (tip 3 μm away from the sample, tip out). The difference of the two spectra (tip in- tip out) indicates the enhancement induced by the metallic tip.

To estimate the TERS contrast of the current experiment, a series of Raman spectra were acquired at selected locations on the surface of the SiNW with an acquisition time of 10 s/spectrum. The TERS spectrum of the silicon nanowires along with the Raman spectra obtained with tip in (near+far field contribution) and tip-out (far-field contribution) of the [100] nanowire are shown in Figure 6. Here, the TERS spectrum is obtained by calculating the difference between the two experiments conducted with the tip close and far from the sample.

As shown in Figure 6, in addition to the improvement of the spatial resolution, a contrast ratio of C ∼ 3–4 was achieved. This value differs from C ∼ 20 obtained in the previous experiment. Such variation may arise from the difference of activity between the different tips used in this study. The observed enhancement originates from the LSPR of the TERS tip which is highly sensitive to the shape and the quality of the metal coating and it is known that in TERS tip reproducibility is critical [39].

**Figure 7.**
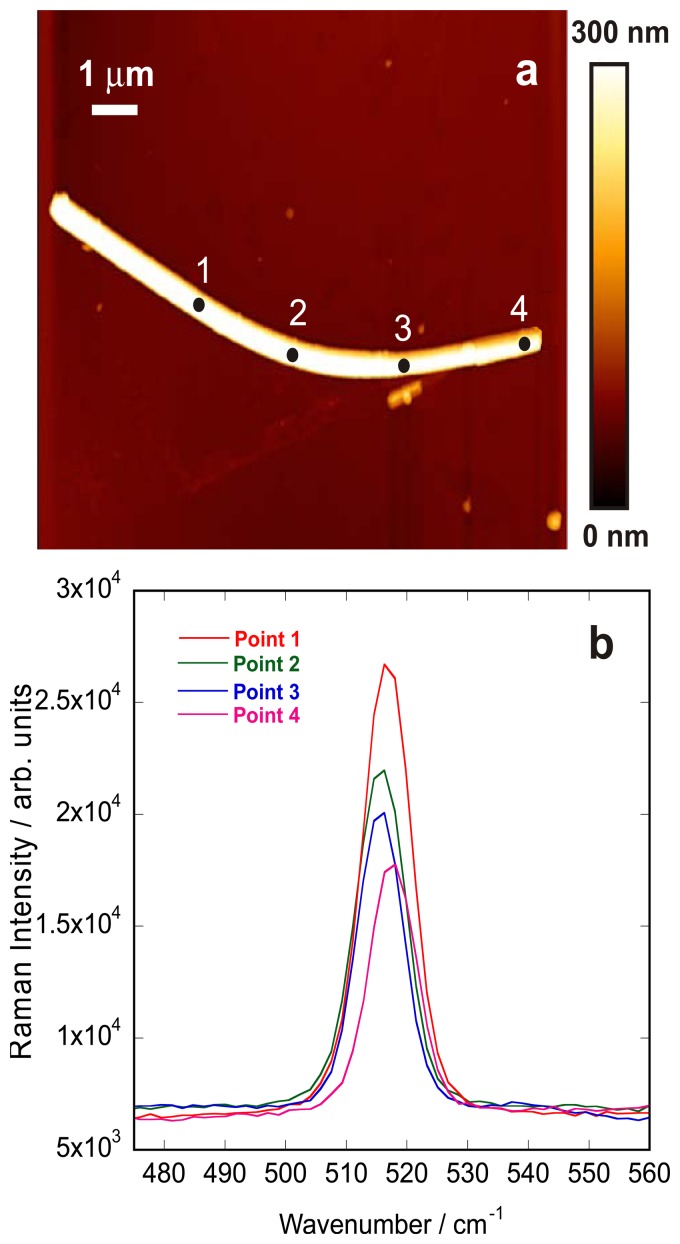
(**a**) AFM image of a bent bundle of silicon nanowires; (**b**) TERS spectra collected along the nanowire in position 1–4.

Additionally, the influence of the lattice strain on vibrational frequency shift and FWHM of silicon Raman signal was studied by TERS. Bending could be considered a source of lattice strain and previous studies have revealed a conductance enhancement in different nanowires as a result of the bending induced strain [40]. We studied the effect of this strain on the first order optical phonon Raman peak of silicon nanowires by collecting the TERS spectra at selected positions over of a bent silicon nanowire. The AFM image of this bent structure is presented in Figure 7a, while Figure 7b shows the TERS spectra obtained from different locations on this structure assigned to the nanostructure variations. The TERS Raman spectra show over 2 cm^−1^ shift towards lower frequencies and a slight broadening for the bent portion of the nanowire (points 2,3) as compared to the straight portions of the single wire (points 1,4). The broadening effect originates from the fact that nanowires experience compressive strain on the inner side of curvature and tensile strain on the outer side [41].

Compression leads to a positive frequency shift of phonon modes, whereas tension induces a negative shift and together, the two produce a broadening [41]. The broadening effect disappears as we move towards the straight ends of the bent wire and the Raman peak appears at 518 cm^−1^ again. This bending strain that causes lattice strain can be defined as D/2R, where D denotes the diameter of the nanowire and R is the radius of the curvature of the bent nanowire [10,40]. Therefore, our results indicate that: (i) the red shift proves that tensile strain has a larger magnitude and that (ii) compressive and tensile strain coupling results in broadening of the peak. Such observations were also seen in ZnO nanowires [42]. For such nanocrystals the compressive and tensile stress are shifting the LO phonon to higher and lower wavenumbers, respectively. These results can also be compared with the work of Zheng *et al.* using high resolution transmission electron microscopy [41]. In their work they evidenced that the neutral-strain axis was shifted from the compressive zone to the tensile region and that significant strain variation along the bending axial direction in the compressive region was revealed. In our experiments we did not measured significant changes of the TERS spectra along the bending direction. In is possible that the particular enhancement along the longitudinal direction with respect to the tip axis may cause some difficulties to provide a quantitative vision of the induced strains in particular using a transmission geometry as opposed to reflection TERS setup.

**Figure 8.**
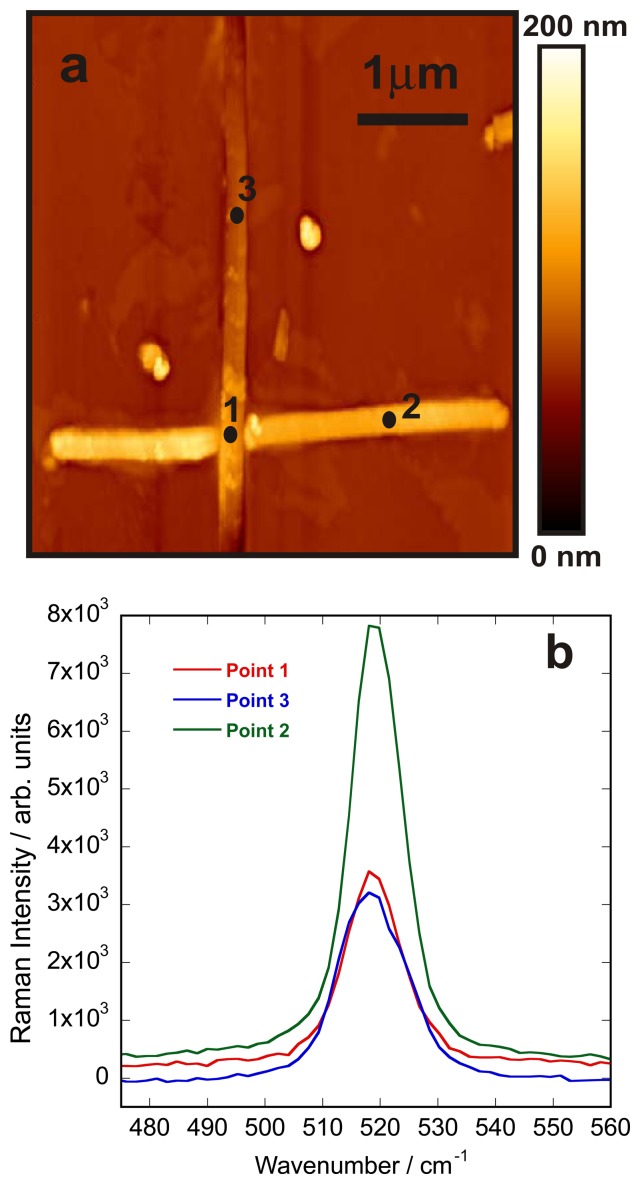
(**a**) AFM image of two crossed silicon nanowires; (**b**) TERS spectra measured on points 1–3.

Finally the spectral variations due to potential strains induced by crossing were investigated. Crossed nanowires play an important role in fabrication of the nanodevices and it has been shown that overlapping crossed silicon nanowires make good electrical contact with each other and create electrical junctions which exhibit good diode behavior [43]. To investigate the presence of strain in the nanowires, the AFM image of arbitrary two crossed nanowires was first obtained (Figure 8a). The TERS spectra of different spots on the crossed wires were then collected and are shown in Figure 8b. The acquisition time was 10 s for each of these spectra.

As it is shown in Figure 8b, the Raman signal of silicon, which appears at around 518 cm^−1^ for a single silicon nanowire, does not experience any significant spectral shift or a change in FWHM, suggesting that no strain is induced in the wire as a result of crossing.

## 4. Conclusions

In this work, TERS of isolated nanowires were investigated using a nanocrystalline diamond tip coated with silver. Despite the low aspect ratio of the AFM tip used in this study, the TERS results show a lateral resolution close to the AFM cross section. We believe that higher aspect ratio AFM tips should lead to much better spatial resolution that should correlate with the diameter of a single nanowire. Since the mechanical, electrical electronic, optical and thermal properties of silicon nanowires are sensitive to the structure of the wires, the TERS technique can provide reliable insights into the influence of morphology on any of these properties.

## References

[1] Liao F., Liu S., Shao M., Lee S.-T. (2009). Surface-dependent chemical properties of silicon nanowires: The acceleration of copper oxidation. Appl. Phys. Lett..

[2] Tsakalakos L., Balch J., Fronheiser J., Korevaar B.A., Sulima O., Rand J. (2007). Silicon nanowire solar cells. Appl. Phys. Lett..

[3] Ge M., Rong J., Fang X., Zhou C. (2012). Porous Doped silicon nanowires for lithium ion battery anode with long cycle life. Nano Lett..

[4] Zhu J., Cui Y. (2010). Photovoltaics: More solar cells for less. Nat. Mater..

[5] Boukai A.I., Bunimovich Y., Tahir-Kheli J., Yu J.-K., Goddard W.A., Heath J.R. (2008). Silicon nanowires as efficient thermoelectric materials. Nature.

[6] Hochbaum A.I., Chen R., Delgado R.D., Liang W., Garnett E.C., Najarian M., Majumdar A., Yang P. (2008). Enhanced thermoelectric performance of rough silicon nanowires. Nature.

[7] Piscanec S., Cantoro M., Ferrari A.C., Zapien J.A., Lifshitz Y., Lee S.T., Hofmann S., Robertson J. (2003). Raman spectroscopy of silicon nanowires. Phys. Rev. B: Condens. Matter Mater. Phys..

[8] Li B., Yu D., Zhang S.-L. (1999). Raman spectral study of silicon nanowires. Phys. Rev. B: Condens. Matter Mater. Phys..

[9] Chen J., Conache G., Pistol M.-E., Gray S.M., Borgstroem M.T., Xu H., Xu H.Q., Samuelson L., Haakanson U. (2010). Probing strain in bent semiconductor nanowires with raman spectroscopy. Nano Lett..

[10] Chen Y., Peng B., Wang B. (2007). Raman spectra and temperature-dependent Raman scattering of silicon nanowires. J. Phys. Chem. C.

[11] Tarun A., Hayazawa N., Balois M.V., Kawata S., Reiche M., Moutanabbir O. (2013). Stress redistribution in individual ultrathin strained silicon nanowires: A high-resolution polarized Raman study. New J. Phys..

[12] Bailo E., Deckert V. (2008). Tip-enhanced Raman scattering. Chem. Soc. Rev..

[13] Kazemi-Zanjani N., Chen H., Goldberg H.A., Hunter G.K., Grohe B., Lagugné-Labarthet F. (2012). Label-free mapping of osteopontin adsorption to calcium oxalate monohydrate crystals by tip-enhanced Raman spectroscopy. J. Am. Chem. Soc..

[14] Pozzi E.A., Sonntag M.D., Jiang N., Klingsporn J.M., Hersam M.C., van Duyne R.P. (2013). Tip-enhanced Raman imaging: An emergent tool for probing biology at the nanoscale. ACS Nano.

[15] Kawata S. (2013). Plasmonics for nanoimaging and nanospectroscopy. Appl. Spectrosc..

[16] Schmid T., Opilik L., Blum C., Zenobi R. (2013). Nanoscale chemical imaging using tip-enhanced Raman spectroscopy: A critical review. Angew. Chem. Int. Ed..

[17] Hartschuh A. (2008). Tip-enhanced near-field optical microscopy. Angew. Chem. Int. Ed..

[18] Gucciardi P.G., Valmalette J.C. (2010). Different longitudinal optical-transverse optical mode amplification in tip enhanced Raman spectroscopy of GaAs (001). Appl. Phys. Lett..

[19] Berweger S., Neacsu C.C., Mao Y., Zhou H., Wong S.S., Raschke M.B. (2009). Optical nanocrystallography with tip-enhanced phonon Raman spectroscopy. Nat. Nanotechnol..

[20] Marquestaut N., Talaga D., Servant L., Yang P., Pauzauskie P., Lagugné-Labarthet F. (2009). Imaging of single GaN nanowires by tip enhanced Raman spectroscopy. J. Raman Spectrosc..

[21] Ogawa Y., Yuasa Y., Minami F., Oda S. (2011). Tip-enhanced Raman mapping of a single Ge nanowire. Appl. Phys. Lett..

[22] Reparaz J.S., Peica N., Kirste R., Goni A.R., Wagner M.R., Callsen G., Alonso M.I., Garrriga M., Marcus I.C. (2013). Probing local strain and composition in Ge nanowires by means of tip-enhanced Raman scattering. Nanotechnology.

[23] Lee N., Hartschuh R.D., Mehtani D., Kisliuk A., Maguire J.F., Green M., Foster M.D., Sokolov A.P. (2007). High contrast scanning nano-Raman spectrosocpy of silicon. J. Raman Spectrosc..

[24] Bouhelier A., Beverluis M.R., Novotny L. (2003). Near-field scattering of longitudinal fields. Appl. Phys. Lett..

[25] Pauzauskie P.J., Talaga D., Seo K., Yang P., Lagugné-Labarthet F. (2005). Polarized Raman confocal microscopy of single gallium nitride nanowires. J. Am. Chem. Soc..

[26] De Wolf I. (1996). Micro-Raman spectroscopy to study local mechanical stress in silicon integrated circuits. Semicond. Sci. Technol..

[27] Cançado L.G., Hartschuh A., Novotny L. (2009). Tip-enhanced Raman spectroscopy of carbon nanotubes. J. Raman Spectrosc..

[28] Stadler J., Schmid T., Zenobi R. (2012). Developments in and practical guidelines for tip-enhanced Raman spectroscopy. Nanoscale.

[29] Novotny L. (2008). Optical antennas tuned to pitch. Nature.

[30] Novotny L., van Hulst N. (2011). Antennas for light. Nat. Photon..

[31] Zhang M.-L., Peng K.-Q., Fan X., Jie J.-S., Zhang R.-Q., Lee S.-T., Wong N.-B. (2008). Preparation of large-area uniform silicon nanowires arrays through metal-assisted chemical etching. J. Phys. Chem. C.

[32] Novotny L., Sanchez E.J., Xie X.S. (1998). Near-field optical imaging using metal tips illuminated by higher-order Hermite-Gaussian beams. Ultramicroscopy.

[33] Hayazawa N., Saito Y., Kawata S. (2004). Detection and characterization of longitudinal field for tip-enhanced Raman spectroscopy. Appl. Phys. Lett..

[34] Anastassakis E., Pinczuk A., Burstein E., Pollak F.H., Cardona M. (1970). Effect of static uniaxial stress on the Raman spectrum of silicon. Solid State Commun..

[35] Lockwood D.J., Baribeau J.-M. (1992). Strain-shift coefficients for phonons in Si1-xGex epilayers on silicon. Phys. Rev. B.

[36] Pashaee F., Hou R., Gobbo P., Workentin M.S., Lagugné-Labarthet F. (2013). Tip-enhanced Raman spectroscopy of self-assembled thiolated monolayers on flat gold nanoplates using Gaussian-Transverse and radially polarized excitations. J. Phys. Chem. C.

[37] Kazemi-Zanjani N., Pashaee F., Lagugné-Labarthet F. (2012). Tip-enhanced Raman spectroscopy: Application to the study of single silicon nanowire and functionalized gold surface. Proc. SPIE.

[38] Birrell J., Gerbi J.E., Auciello O., Gibson J.M., Johnson J., Carlisle J.A. (2005). Interpretation of the Raman spectra of ultrananocrystalline diamond. Diamond Relat. Mater..

[39] Asghari-Khiavi M., Wood B.R., Hojati-Talemi P., Downes A., McNaughton D., Mechler A. (2012). Exploring the origin of tip-enhanced Raman scattering; preparation of efficient TERS probes with high yield. J. Raman Spectrosc..

[40] Han X., Jing G., Zhang X., Ma R., Song X., Xu J., Liao Z., Wang N., Yu D. (2009). Bending-induced conductance increase in individual semiconductor nanowires and nanobelts. Nano Res..

[41] Zheng K., Han X., Wang L., Zhang Y., Yue Y., Qin Y., Zhang X., Zhang Z. (2009). Atomic mechanisms governing the elastic limit and the incipient plasticity of bending Si nanowires. Nano Lett..

[42] Yan B., Chen R., Zhou W., Zhang J., Sun H., Gong H., Yu T. (2010). Localized suppression of longitudinal-optical-phonon–exciton coupling in bent ZnO nanowires. Nanotechnology.

[43] Cui Y., Lieber C.M. (2001). Functional nanoscale electronic devices assembled using silicon nanowire building blocks. Science.

